# Decidual cells and decidualization in the carnivoran endotheliochorial placenta

**DOI:** 10.3389/fcell.2023.1134874

**Published:** 2023-03-16

**Authors:** Mónica Elizabeth Diessler, Rocío Hernández, Gimena Gomez Castro, Claudio Gustavo Barbeito

**Affiliations:** ^1^ Laboratorio de Histología y Embriología Descriptiva, Experimental y Comparada (LHYEDEC), Facultad de Ciencias Veterinarias, Universidad Nacional de La Plata (FCV, UNLP), La Plata, Argentina; ^2^ Consejo Nacional de Investigaciones Científicas y Técnicas (CONICET), FCV, UNLP, La Plata, Argentina

**Keywords:** placenta, decidual, endotheliochorial, carnivore, feline, canine, DSCs

## Abstract

Decidualization is considered a distinctive feature of eutherian pregnancy, and has appeared during evolution along with the development of invasive forms of placentation, as the endotheliochorial placenta. Although decidualization is not massive in carnivores, as it is in most species developing hemochorial placentas, isolated or grouped cells regarded as decidual have been documented and characterized, mainly in bitches and queens. For the majority of the remaining species of the order, data in the bibliography are fragmentary. In this article, general morphological aspects of decidual stromal cells (DSCs), their time of appearance and lasting, data about the expression of cytoskeletal proteins and molecules considered as markers of decidualization were reviewed. From the data reviewed, it follows that carnivoran DSCs take part either in the secretion of progesterone, prostaglandins, relaxin, among other substances, or at least in the signaling pathways triggered by them. Beyond their physiological roles, some of those molecules are already being used, or are yet under study, for the non-invasive endocrine monitoring and reproductive control of domestic and wild carnivores. Only insulin-like growth factor binding protein 1, among the main decidual markers, has been undoubtedly demonstrated in both species. Laminin, on the contrary, was found only in feline DSCs, and prolactin was preliminary reported in dogs and cats. Prolactin receptor, on the other hand, was found in both species. While canine DSCs are the only placental cell type expressing the nuclear progesterone receptor (PGR), that receptor has not been demonstrated neither in feline DSCs, nor in any other cell in the queen placenta, although the use of PGR blockers leads to abortion. Against this background, and from the data gathered so far, it is unquestionable that DSCs in carnivorans do play a pivotal role in placental development and health. The knowledge about placental physiology is critical for medical care and breeding management, primarily in domestic carnivores; it is also absolutely crucial for a conservation approach in the management of endangered carnivore species.

## 1 Introduction

Placentation in mammals appeared as a single event, which occurred before the divergence of Theria into Metatheria and Eutheria. The tight contact between maternal tissues and fetal trophoblast (TB), however, is only typical of the eutherian line (Wagner et al., 2014); morphological and molecular studies carried out in the last decades agree on the invasive nature of eutherian basal placenta (Carter, 2001; Wildman et al., 2006; Mess and Carter, 2007). In fact, according to the most recent transcriptomic analysis, it is more likely that it was hemochorial (Mika et al., 2022). As it is reviewed in Wagner et al. (2014), along with an invasive placentation, the origin of decidual cells -and the early recognition of pregnancy with long-lasting luteal progesterone production-were the three evolutionary innovations already present in stem eutherians. Development of decidual cells is, therefore, a distinct feature of eutherian pregnancy, absent in placental marsupials and Squamata (Wagner et al., 2014).

The transcriptome of eutherian endometrial stromal cells, when compared to that in marsupials and reptiles, shows a loss or lower expression of genes involved in inflammation, immune response, and resistance to tissue invasion; conversely, genes related to cell cycle progression, proliferation, and insensitivity to estrogens at the beginning of pregnancy show higher expression in eutherians (Kin et al., 2016; Marinić et al., 2021). Those findings are in agreement with the proven roles of decidual cells in the regulation of the local immune response and the prevention of excessive trophoblast invasion (Chavan et al., 2016). Although an inflammatory process occurs in the endometrium of all viviparous mammals at implantation, the proinflammatory cytokine IL17 was not found at that stage in the uterus of any of the three major eutherian clades, unlike what was demonstrated in marsupials. This cytokine recruits neutrophils to the area where it is expressed, among other functions (Chavan et al., 2021).

Although decidualization is triggered by the presence of the conceptus, it can also occur in the non-pregnant uterus in catarrhines primates, some chiropters, the elephant shrew (Emera et al., 2012b) and the spiny mouse (Bellofiore et al., 2021). This pregnancy-independent decidualization occurs during the luteal phase of the cycle and leads ultimately to the recurring partial shedding of the endometrium, that is, to menstruation (Jarrell, 2018).

Considering the association between trophoblast invasiveness and decidualization of the stroma, it is understandable that the latter does not usually occur in species with non-invasive placentas. These variants appeared later in the evolution of eutherian mammals, along with pregnancy lengthening and birth of precocial offspring (Mess and Carter, 2006). In ungulates and cetaceans, without close contact at the maternal-fetal interface, decidual cells have not been found (Wooding and Burton, 2008), to such an extent that gene expression of *Bos taurus’* uterine fibroblasts is more comparable to that in marsupials than to gene expression in the eutherians with invasive placentas (Kin et al., 2016). It was considered for some time that the mare was an exception, based on Amoroso´s references to the endometrial cups as being composed of decidual cells (Amoroso, 1950; Amoroso, 1959). Given that, W.R. Allen wrongly concluded that the equine chorionic gonadotropin (eCG) produced by the cups was maternal, despite it being named as “chorionic”; in a later work of his own, the trophoblastic nature of those cells, and, therefore, the fetal origin of eCG, was demonstrated (reviewed in Antczak et al., 2013). In the ewe, during placentation, the uterine luminal epithelium is eroded and fuses with trophectoderm cells. This higher degree of invasiveness as compared to the remaining ungulates is associated with differentiation of endometrial stromal cells, which has been regarded as a decidualization-like process (Johnson et al., 2003).

The expressions “decidua” and “decidual”, used in comparative placental studies for more than 150 years, have given rise to misinterpretations, as they may describe species, placentas, specific regions in the endometrium, as well as cells. Furthermore, the discovery of several types of uterine stromal cells (which have not been studied in species with endotheliochorial placentas so far) leads to a confusing designation of the endometrial cells undergoing decidualization (Ruiz-Magaña et al., 2022). The term “decidual” was used, since the xix century, to refer to the tissues shed during birth in many eutherians, and led to an obstetric classification of placentas into decidual and non-decidual ones (Huxley, 1864). This decidual tissue is the modified endometrial stroma (Wooding and Burton, 2008).

Decidualization currently refers to a hormone-dependent process by which endometrial stromal fibroblasts differentiate into the so-called decidual stromal cells (DSCs) (Wooding and Burton, 2008; Chavan et al., 2016). This process involves reprogramming of endometrial fibroblasts gene expression with epigenetic modifications (Tamura et al., 2014). It leads to the activation of the progesterone receptor pathway, and a PGE2-dependent activation of PKA pathway (Erkenbrack et al., 2018). This genetic reprogramming generates morphological and functional changes such as larger size, acquisition of a round shape, ability to store glycogen and lipids (Chavan et al., 2016), as well as the expression of different substances considered as decidual markers, such as prolactin receptor and IGFBP-1 (Dunn et al., 2003; Aghajanova et al., 2010). In addition to the transformation of endometrial fibroblasts into decidual cells, modification of the extracellular matrix (ECM), vascularization, and in some species, appearance of uterine natural killer cells, also occur (Wooding and Burton, 2008).

DSCs have been related not only to the successful establishment of the pregnancy but also to its maintenance over time; however, in many species much of pregnancy takes place with little or no DSCs. Several results support the hypothesis that the decidualization that develops at implantation and lasts only early pregnancy is the basal state in eutherians, as it has been reported in species of Xenarthra (Dasypus: Chavan and Wagner, 2016; Chavan, 2018), Afrotheria (Procavia: Thursby-Pelham, 1925; Chavan et al., 2016; Echinops: Carter et al., 2004; 2005) and Boreoeutheria (hedgehog: Chavan et al., 2016). In these species, there is a limited decidual reaction mainly during implantation, the decidual tissue gradually thins out and it disappears at advanced pregnancy stages. In some Boreoeutherians, decidualization is prolonged during gestation, although decidual cell number frequently decreases along pregnancy (Chavan et al., 2021).

Endotheliochorial placentas are typical of species grouped in the three abovementioned clades. In regard to decidualization in endotheliochorial placentas, the presence of decidual cells as a state of the character “differentiation of endometrial stroma” is considered typical of several genera developing this type of maternal-fetal interface. For instance, it was reported in Sorex, Suncus and Talpa (shrews and moles; Boreoeutheria, Laurasiatheria, Eulipotyphla), Bradypus (sloths; Xenarthra), Micropotamogale and Trichechus (otter shrews and manatee, respectively; Afrotheria) (Mess and Carter, 2006; Carter and Enders, 2010; Ferner et al., 2014). Despite this state was not reported as typical of any genera comprised in Carnivora (Carter and Mess, 2017), isolated or grouped cells regarded as decidual have been documented and characterized in carnivores (citations registered in section 2).

Most carnivores develop an endotheliochorial interface in their definitive placenta (with or without syncytial trophoblast), the exception being the hyena (*Crocuta crocuta*) that has a hemochorial placenta with decidual cells. However, early stages of placental formation have not been studied; it is not known whether or not the hemochorial condition is preceded by an endotheliochorial condition (Enders et al., 2006; Wooding and Burton, 2008).

Decades ago, Mossman stated that, as nothing was known about the function of these cells in carnivores and they were not in the position of the “typical” decidua, naming them “decidual” was unfortunate and they must be regarded as “maternal giant cells” (Mossman, 1987). That expression does not reflect the aspect of decidual cells in bitches, which are not giant; besides, vast knowledge has been gained so as to currently refer to them as decidual stromal cells (DSCs), which is the acronym chosen in this review.

## 2 DSCs in Carnivora

### 2.1 Cytoskeletal proteins and origin of DSCs

Several works dealt with aspects of the cytoskeleton phenotype of DSCs; they contributed to the knowledge of both their origin and the value of certain cytoskeletal molecules as decidual markers. Despite their epithelioid appearance, decidual cells are derived from mesenchymal cells of the uterine stroma. As such, they are negative for cytokeratins and positive for vimentin. It was reported that vimentin expression, related to total protein expression, remains unaltered during decidualization in rats (Glasser and Julian, 1986); however, a change in the packaging and position of the vimentin intermediate filaments was observed in mice (Oliveira et al., 2000). Although the protein alpha smooth muscle actin (α-SMA) was considered a marker of human decidual cells (Oliver et al., 1999), recently it was stated that its gene (*ACTA2*) is expressed in endometrial stromal fibroblasts in a pre-DSC state, named by the authors as “activated endometrial stromal fibroblasts”, rather than in a canonical decidual state (Stadtmauer and Wagner, 2022).

Desmin protein was detected in DSCs in mice, rats, humans, among others. It has been shown that desmin is selectively induced in the rat decidualizing stroma (and it is copolymerized with vimentin within the same intermediate filaments) compared to its hormonally sensitized but yet non-decidualized counterpart, where it was not -or barely-detected (Glasser and Julian, 1986). In addition to humans and rodents, co-expression of vimentin and desmin in intermediate filaments was also found in the bat *Carollia perspicillata* (Rasweiler et al., 2000).

Ever since then, desmin has been widely reported as a marker for the decidual differentiation in other rodents, bats, and humans (Oliveira et al., 2000; Rasweiler et al., 2000). Can et al. (1995) regarded desmin expression in human decidual cells as “temporary”, attributing this early switch-on of desmin synthesis to the structural plasticity of stromal cells during decidualization. He stated that desmin protein expression gradually decreases (Can et al., 1995); the same tendency was verified in mice and rats, to such an extent that in advanced pregnancy desmin could not be detected (Glasser and Julian, 1986; Oliveira et al., 2000).

Over the years, stromal cell identity and origin during decidualization have been discussed, and the use of different terms to refer to the same cells resulted in considerable misunderstandings (Ruiz-Magaña et al., 2022). The existence of stromal resident progenitor cells in the perivascular niche of the human endometrium has been documented years ago and extensively studied (revised in Gargett et al., 2016). Later, an equivalent cell population in mice was identified and characterized; besides, different subpopulations of endometrial fibroblasts and perivascular cells were described in this species (Kirkwood et al., 2021). Several features of decidual cells, such as expression of α-SMA, in addition to their contractile capacity, led to consider that they were related to myofibroblasts and pericytes (Oliver et al., 1999; Muñoz-Fernández et al., 2018; Ruiz-Magaña et al., 2022). Whereas α-SMA presence is the defining property of myofibroblasts in all tissue types, and thus a reliable marker of myofibroblast differentiation (Watsky et al., 2010), desmin is not a feature of these cells (Eyden, 2008). On the other hand, pericytes express vimentin, α-SMA and desmin, although there may be a shift between desmin (+) and (−) cells, and some heterogeneity among subtypes of pericytes (reviewed in Zhu et al., 2022). As many similarities have been found between endometrial pericytes and predecidual stromal cells in humans, they are proposed to be the same cell (Muñoz-Fernández et al., 2018).

**FIGURE 1:**
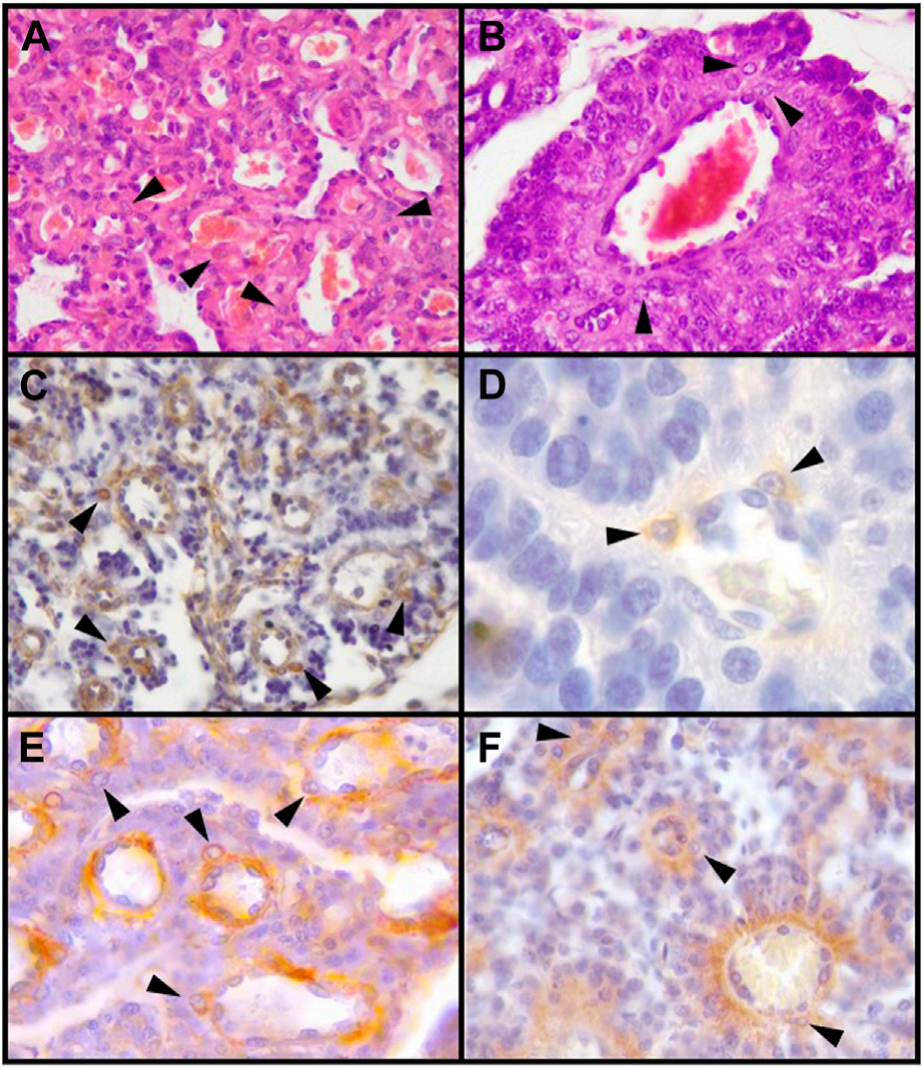
Decidual stromal cells (DSCs) in the canine placenta. Histological (HE) and immunohistochemical staining. **(A, B)**. HE. **(A)** 10X; **(B)** 40X. **(C)**. Vimentin (Clone V9, RTU, Code: IS630. Dako) (10X). **(D)**. Desmin (Clone D33, RTU, Code: IS606. Dako) (100X). **(E)**. α-SMA (Code: NCL-L-SMA. Novocastra) (40X). **(F)**. IGFBP-1 (40X) (Code: PAAH1. Novozimes GroPep Ltd.). Black arrowheads: DSCs. IHC. DAB. Hernández et al., 2017.

The mesenchymal origin of the decidual cells is also confirmed in carnivores; as in other species, DSCs of bitches and queens are negative for cytokeratins and positive for vimentin, both *in vivo* and *in vitro* (Walter and Schönkypi, 2006; Fernández et al., 2014; Kautz et al., 2015; Schäfer-Somi et al., 2015; Graubner et al., 2017) (Figures 1, 3A). This basic profile is certainly shared by the non-decidualized endometrial stromal cells. The epithelioid appearance, and the upregulation of some ECM or cell-to-cell adhesion proteins *in vitro* DSCs might indicate some degree of mesenchymal-epithelial transition of these cells, although retaining the cytokeratin-vimentin pattern of mesenchymal lineage (Graubner et al., 2020; Kazemian et al., 2022; Tavares Pereira et al., 2022). The protein α-SMA is not expressed or only weakly present in uterine stromal cells of non-pregnant bitches and in pregnant ones at the preimplantation stage. Following placentation, or *in vitro* decidualization, its expression markedly increases (Kautz et al., 2015; Graubner et al., 2018). In the cat, α-SMA has been detected in decidualized cells both in the labyrinth and in the junctional zone (Walter and Schönkypl, 2006; Hernández et al., 2017) (Figures 1E, 3B–D). In minks and Southern elephant seals perivascular cells share this pattern (see 2.2) (Winther H et al., 1999; Diessler et al., 2020). DSCs of the bitch were also positive for desmin (Vermeirsch et al., 2000; Hernández et al., 2017) (Figure 1), whereas in the queen DSCs were negative, at least in >45 days post coitum (dpc) placentas (Fernández et al., 2014). In view of the dynamics of desmin staining in humans and rodents, this result in late placentas might obey to a decrease in desmin expression with time (Fernández et al., 2014). Future studies may determine whether or not DSCs are related to pericytes in these species.

### 2.2 Decidual cells in Caniformia

Within this suborder, microscopical studies of the placenta of seven families have been published. There are reports on Ailuridae (Benirschke, 2011), Mustelidae (Rau, 1925; Wislocki and Amoroso, 1956; Lawn AM and Chiquoine AD, 1965; Pfarrer et al., 1999; Winther H et al., 1999; Lindeberg, 2008), Otariidae and Phocidae (Harrison and Young, 1966; Sinha and Erickson, 1974; Diessler et al., 2020; Gomez Castro et al., 2022b; Hernández et al., 2022), Procyonidae (Creed and Biggers, 1963; Favaron et al., 2014), Ursidae (Rau, 1925; Young, 1969; Wimsatt, 1974), and Canidae (Kehrer, 1973; Zybina et al., 2001; main citations about *Canis lupus* familiaris are registered in Section 2.2.1).

Only in the American mink (*Neogale vison*, formerly *Mustela vison*) and in the Southern elephant seal (*Mirounga leonina*), vimentin and α-SMA positive periendothelial cells have been reported (Winther H et al., 1999; Diessler et al., 2020).

In microscopic descriptions of *Ictonyx striatus* (striped polecat, formerly *Zorrilla striata*), *Procyon Lotor* (raccoon), *Gulo gulo luscus* (wolverine), *Ursus americanus* and *Ursus arctos* (American black bear and brown bear, respectively), and in an ultrastructural study in *Mustela putorius furo* (ferret), it is explicitly stated that their placentas lack giant cells as those found in cats (Rau, 1925; Wislocki and Amoroso, 1956; Creed and Biggers, 1963; Lawn AM and Chiquoine AD, 1965; Wimsatt, 1974). Many other articles do not mention decidual cells whatsoever.

Leaving aside *Canis lupus familiaris,* and among other canid species, there are only a few reports of placental microscopical features. Therefore, in this review we use expressions such as “canine placenta” to refer specifically to the placenta of the bitch. In the silver fox, Zybina et al. mentioned a few round stromal cells to which they referred as “probably decidual” (Zybina et al., 2001). Numerous giant cells compressed between the chorionic leaves were described in a fox (Kehrer, 1973).

#### 2.2.1 DSCs in *Canis lupus familiaris*

##### 2.2.1.1 Discovery and characterization

Although the functional differentiation of the bitch uterus following implantation has been recognized around the mid XIX century, the actual finding of decidual cells occurred many years later. Wynn and Corbett, in 1969, affirmed after an ultrastructural study that giant cells of endometrial origin were absent in the canine placenta (Wynn and Corbett, 1969). Just a few months after, John Anderson, rectifying a preliminary study of his own authorship, mentioned that dog and cats were similar regarding the persistence of maternal decidual cells, a finding that he characterized as “unsuspected” (Anderson, 1966; Anderson, 1969). However, he also stated that they could not be account unequivocally as maternal, and that they were impossible to identify in paraffin sections. For more than 30 years after Anderson’s work, only rare mentions of decidual cells, or inconclusive findings concerned with their existence in this species, were published. Afterward, more advanced techniques and growing knowledge allowed Vermeirsch et al. (2000) to report that those cells could be referred to as decidual cells. It was not until a little over the last decade when those cells were undoubtedly evidenced at a structural level and characterized in regard to several morphological and biochemical aspects, described below. Also, physiological implications of those observations began to be elucidated.

Along with the data obtained from immunohistochemical and molecular analyses of placental tissue *in vivo*, a substantial contribution to the knowledge about canine DSCs comes from the *in vitro* decidualization of canine uterine stromal cells (Kautz et al., 2015), and from the establishment of an immortalized cell line from those cells (Graubner et al., 2017).

In an ultrastructural study, decidual cells were reported as present and mitotically active as early as 16 dpc, before placentation takes place (Barrau et al., 1975). In HE stained specimens, morphological differentiation of stromal endometrial cells (namely, rounding and enlarging) was reported from 17 dpc, being the changes in genetic expression induced by free-floating embryos (Graubner et al., 2017).

Besides the identification of DSCs and the characterization by their cytoskeletal proteins, many other molecules have been proven to be strongly induced or, at least, upregulated in canine DSCs, compared to their precursors. Some of them are hormone receptors, as prolactin receptor (PRLR), progesterone receptor (PGR), estrogen receptor α (ERα), and oxytocin receptor (OTR). Other molecules detected in DSCs are insulin-like growth factors (IGFs), IGF binding protein 1 (IGFBP-1), and PTGES (Kautz et al., 2014; Kautz et al., 2015; Graubner et al., 2017; Hernández et al., 2017) (Figure 1F). Conversely, although targeted to the canine placenta as a whole, other substances were not significantly labeled in decidual cells. That is the case of prolactin (PRL), vascular endothelial growth factor A (VEGF-A), gonadotropin releasing hormone receptor (GnRH-R), glucocorticoid receptor (GR/NR3C1), orexin A (OXA), orexin type 2 receptor (OX2R), leptin, e-cadherin, β-catenin, FN1, TIMP2 (Dall’Aglio et al., 2014; Kautz et al., 2014; Kautz et al., 2015; Balogh et al., 2015; Schäfer-Somi et al., 2015; Gram et al., 2016; Payan-Carreira et al., 2016; Graubner et al., 2018) (Table 1).

**TABLE 1:** Proteins detected in carnivoran DSCs *in vivo.*

	Canine DSCs	Feline DSCs
Cytokeratins	—^a^	—
Vimentin	+^b^	+
α-SMA	+^b^	+
Desmin	+^c^	—
PRL	+^d^	+^d^
PRLR	+	+^d^
PGR	+	—
ERα	+	—
OTR	+	
RLN	—	+^e^
RXFP2	+	
GnRH-R	-/weak	
GR/NR3C1	-/weak	
OXA	—	+
OX1R		+
OX2R	—	—
Leptin/LEPR	weak	+
PTGS2	-/weak	+
PGFS		+
IGFs	+	
IGFBP-1	+	+
EGF/EGFR		+
TGFα		+
VEGF-A/R-2		+^d^
e-cadherin	—	
β-catenin	—	
FN1	weak	
TIMP2	weak	
Laminin	—	+
MMP2		+
MMP1,9,13		—
Galectins		Gal 1, 3, 9 +
transporters: GLUT1/3; AQP2/AQP8		—

α-SMA, smooth muscle actin; AQPs, aquaporins; EGF/EGFR, epidermal growth factor/receptor; ERα, estrogen receptor alpha; FN1, fibronectin; GLUT, glucose transporter; GnRH, gonadotropin releasing hormone; GR/NR3C1, glucocorticoid receptor; IGFBP-1, IGF binding protein 1; IGFs, insulin-like growth factors; LEPR, leptin receptor; MMPs, metalloproteinases; OTR, oxytocin receptor; OX1R, orexin type 1 receptor; OX2R, orexin type 2 receptor; OXA, orexin A; PGR, progesterone receptor; PRL, prolactin; PRLR, prolactin receptor; PGFS, prostaglandin F2α—synthase; PTGES, prostaglandin E synthase; RLN, relaxin; RXFP2, the relaxin family peptide receptor 2; TGFα, transforming growth factor alpha; TIMP2, tissue inhibitor of metalloproteinase 2; VEGF-A/R-2, vascular endothelial growth factor A and receptor 2.

^a^ Also negative in Southern elephants.

^b^ Also positive in American minks and Southern elephants.

^c^ Negative in Southern elephants.

^d^ Preliminary study.

^e^ Also positive in Iberian lynxes.

Trophoblast and maternal decidual cells are embedded in the ECM at the maternal-fetal interface, and ECM might be involved in decidual cell avoidance of trophoblast invasion. It has been proven that murine and human decidual cells *in vitro* secrete ECM components as entactin, fibronectin, type IV collagen, heparan sulphate, and laminin, being the last one a decidual marker (Wewer et al., 1986; Dunn et al., 2003). Regarding canine DSCs, some molecules targeted to them as FN-1 or TIMP2 were only weakly labeled, and laminin could not be evidenced (Graubner et al., 2018). ECM1, *COL4A1* transcripts abundance was increased during *in vitro* decidualization; that rise was blocked by the action of antigestagens (Kazemian et al., 2022; Tavares Pereira et al., 2022).

From the abovementioned findings, some speculations have been made and some uncertainties remain; they are discussed below within the framework of their relevance to some biological processes peculiar or relevant to canine reproduction.

##### 2.2.1.2 Steroidogenesis and signaling through progesterone (P4)

One of the peculiar features of canine reproduction is that no placental steroidogenesis has been detected in this species. Progesterone, therefore, is exclusively secreted by luteal cells (reviewed in Kowalewski et al., 2021). Since Vermeirsh et al., 2000 reported that DSCs are the only cells in the canine placenta expressing the progesterone receptor it became increasingly clear their crucial role in maintaining canine pregnancy (Vermeirsch et al., 2000). In their work the results are expressed in terms of positivity for ER and PGR in “desmin positive cells”.

The receptor identified is PGR, a member of the nuclear receptor superfamily of transcription factors; upon binding P4, it dimerizes, enters the nucleus and binds DNA (Wetendorf and DeMayo, 2012). PGR gene is expressed in pregnant uteri as early as at the preimplantation stage (10 dpc) (Schäfer-Somi et al., 2008; Kowalewski et al., 2010) and it decreases onwards (Kowalewski et al., 2010). P4 induces endometrial stromal cell differentiation, and influences the cycle, survival, junctions, and secretory activity of the cell, by activating and repressing a multitude of gene pathways (Wetendorf and DeMayo, 2012; Patel et al., 2015). In addition to the classical signaling pathway, it is also known that P4 exerts several additional effects through membrane receptors, or through the monomeric form of PGR (which exert, initially, non-nuclear actions). Progesterone has also receptor-independent actions, e.g., the regulation of cholesterol metabolism (revised in Bishop, 2013). Canine DSCs also express, to a lesser extent, ER-a (Vermeirsch et al., 2000; Graubner et al., 2017).

##### 2.2.1.3 DSCs and prepartal luteolysis: Oxytocin and prostaglandins

Other foci of the research on canine reproductive physiology have been the mechanism of prepartal luteolysis, and the role of decidual and trophoblast cells regarding that matter. On these topics, molecules related to oxytocin signaling and prostaglandin synthesis, and their relation to serum P4 levels, have been studied (Kowalewski et al., 2010; 2020; 2021; Kowalewski, 2012; Gram et al., 2014a; Gram et al., 2014b). Given the similarities found between natural prepartal and antigestagen-induced luteolysis, the pivotal involvement of DSCs in this process, as the only cell target for P4 within the labyrinth, was inferred (Kowalewski et al., 2010; Nowak et al., 2019).

In non-pregnant bitches, a gradual luteal regression occurs, as they lack a classical mechanism of maternal recognition of pregnancy, e.g., the luteolytic action of uterine PGF2-α as ewes, cows, does, sows, and mares, among other females, have (Kowalewski et al., 2015; Noakes et al., 2018). However, in pregnant bitches, prepartal luteolysis does depend on upregulation of the prostaglandin system, in the scenario of the withdrawal of P4. Paracrine crosstalk between DSCs and trophoblast cells induces that crucial prepartum PGF2-α release (Kowalewski, 2012; Kowalewski et al., 2020).

The expression of OTR in the placenta has been evaluated to better understand endocrine mechanisms leading to PGF2-α output near term. Placental OTR was detected in DSCc both at protein and mRNA levels, being its signal markedly stronger during the prepartum luteolysis (Gram et al., 2014a). This higher availability of OTR near term might obey to the steep decline in P4 and the changes in the cell´s plasma membrane triggered by that decrease (Gimpl and Fahrenholz, 2001; Bishop, 2013). OTR is a membrane bound G-protein coupled receptor (GPCR), although most of it is internalized upon ligand-binding (this might be one plausible reason for its usual cytoplasmic immunolabeling) (Gimpl and Fahrenholz, 2001). High P4 levels may block OTR signaling by several mechanisms, namely, the binding to a membrane protein that interacts with the receptor, or the alteration in cholesterol-phospholipid ratio induced by progesterone (Bishop, 2013). Cholesterol abundance in the plasma membrane is inversely proportional to P4 levels; this is partly due to the non-genomic effect of P4 in triggering a state of cholesterol auxotrophy, that is, the cell inability to synthesize cholesterol, by blocking its intracellular trafficking (Gimpl and Fahrenholz, 2001; Bishop, 2013). Therefore, during placental development (under high P4 levels) membrane cholesterol is low. As cholesterol enhances oxytocin high affinity binding to OTR, downstream signaling is reduced at that stage. Conversely, near term, coinciding with P4 withdrawal, plasma membrane cholesterol and oxytocin-OTR binding increase, signaling downstream OTR leads to phospholipase C activation, rise in arachidonic acid and, finally, prostaglandin synthesis (Gimpl and Fahrenholz, 2001).

In relative gene expression studies of the bitch placenta, it was shown that *PTGS2* (that is, the gene for prostaglandin-endoperoxide synthase 2, the common prostaglandin precursor synthase, also known as COX2) and *PTGES* (gene for PGE2 synthase) were increased towards prepartum, whereas *PGFS* (gene for PGF2-α synthase) reached its peak at mid-gestation and decreased afterward. Given that decrease, the abundance of prepartal PGF2-α might obey to the fact that it can also be produced by alternative pathways. PGFS and PTGES mRNA, and PTGS2, were strongly labeled in trophoblast cells. Decidual cells, however, only showed weak or sporadic signals (Kowalewski et al., 2010; Gram et al., 2014b; Kautz et al., 2014). During *in vitro* decidualization, however, PTGS2 and PTGES were upregulated and clearly detected in DSCs (Kautz et al., 2015). In humans OTR and prostaglandins were both detected in decidual cells (Wilson et al., 1988).

##### 2.2.1.4 Prolactin and prolactin receptor

The decidua produces a prolactin-like molecule, named simply as prolactin as it is indistinguishable from pituitary prolactin in humans, although it is somewhat dissimilar in rats (Freeman et al., 2000). It binds to PRLR, and its secretion is locally regulated. Some of the potential roles of placental prolactin are its involvement in the regulation of angiogenesis, apoptosis, immune status, and the stimulation of TB invasion (Rana et al., 2022). In the bitch, prolactin (mainly pituitary prolactin) is considered to be luteotropic (Kowalewski, 2012). Although prolactin is known to be the strongest decidualization marker in humans and rodents (Dunn et al., 2003), it is frequently below detection limits in canine decidual cells. The evolution of regulatory mechanisms of prolactin gene (*Prl*) expression, mainly by transposable-elements, has been studied in humans, orangutans, Old and New World monkeys, tarsiers, mice, rabbits, pigs, dogs, armadillos, and elephants (Emera et al., 2012a; Emera and Wagner, 2012). Emera et al. have concluded that endometrial *Prl* expression is not a shared derived character of all placental mammals, but rather it is a case of convergent evolution of gene expression, as it evolved independently in several eutherian lineages; this might explain the diversity between primates and carnivores. It has been demonstrated that *Prl* was independently recruited into uterine expression in primates, mice and elephants, while it is not expressed in rabbits, pigs, dogs or armadillos (Emera et al., 2012a). The study of evolution of human *dPrl* promoter allowed the authors to hypothesize that the upregulation of the expression of prolactin was a maternal response to the interstitial invasion which evolved in the stem lineage of apes (Emera and Wagner, 2012).

PRL mRNA was investigated in canine placenta homogenates, and no statistical differences were found between non-pregnant and pregnant bitches in this regard, being PRL generally expressed at a very low level (Kautz et al., 2014). *In vitro* decidualized cells also exhibited very low expression of *PRL* (Kautz et al., 2015), even after having achieved high intracellular cAMP concentrations, which appears to be required to obtain maximal PRL expression under the influence of progestins (Dunn et al., 2003). No data regarding immunohistochemical analysis of PRL in decidual cells has been communicated so far, although endometrial stromal cells were immunostained for PRL in a preliminary study (Hernández et al., 2019).

Contrary to the shortage in PRL placental expression, it was reported that PRLR is early induced in the canine placenta by free floating embryos, around 10–12 dpc (Kautz et al., 2014). After *in vitro* decidualization, PRLR mRNA was highly upregulated (Kautz et al., 2015; Graubner et al., 2017). The highly upregulated expression of this receptor, together with the low expression of its ligand might constitute a feature of a species-specific regulatory mechanism (Kautz et al., 2015).

##### 2.2.1.5 Relaxin and relaxin receptors

Relaxin (RLN) is a polypeptide hormone secreted by the trophoblast in the canine placenta, and it is, so far, the only available marker of pregnancy in the dog (Nowak et al., 2017; Nowak et al., 2018). This molecule signals through the relaxin family peptide receptors 1 and 2, (RXFP1, RXFP2), two GPCRs. Both its intracellular signaling pathways and the physiological functions differ widely across a variety of tissues and cell types (Sherwood, 2004; Valkovic et al., 2019). In the canine placenta, relaxin labeling was targeted mainly to cytotrophoblast cells. Nowak et al.´s article shows positivity for RXFP1 receptor in the preimplantation endometrium. After placentation, maternal endothelium was strongly reactive to this receptor, whereas the cytotrophoblast cells were slighter stained. Decidual cells reacted to RXFP2 (Nowak)^1^. mRNA expression of both receptors in the placenta decreased toward term.

The biological significance of RXFP1 expression in endometrial stromal cells and of RXFP2 expression in the decidual cells, with its highest concentration during preimplantation and mid-gestation stages, may be related to the decidualization process. In human endometrial stromal cells (ESCs), sustained cAMP activity induced by relaxin mainly through RXFP1 is associated with decidualization, as it is inferred from the increase in human decidualization markers such as prolactin (Bartsch et al., 2004). There are some differences, though, in the mechanisms by which each receptor triggers cAMP, and the length of the response; in addition, RXFP2 may also bind INSL3 (Halls et al., 2006). Besides the role in decidualization, other local actions of relaxin around term, and its relation to/with P4 levels and the eventual prolactin increase in canine decidual cells were not established yet.

### 2.3 Decidual cells in Feliformia

With the exception of the hyenas, feliforms develop endotheliochorial placentas; up to our knowledge, they have been described only in members of the Felidae family. Moreover, as there are only a few descriptions of placentas in species other than *Felis catus*, all the findings will be described in the Section 2.3.1. From the section title on, the queen´s placenta will be qualified as “feline”.

#### 2.3.1 Feline DSCs

##### 2.3.1.1 Discovery and characterization

Half way through the 20th century, Wislocki and Dempsey for the first time regarded the giant cells in the cores of the lamellae as “*decidual in nature and of maternal origin*”, mainly based on histochemical reactions (Wislocki and Dempsey, 1946). This influential paper contradicted the previous opinion given by Otto Grosser who, though referring to those cells as decidual-like, considered them as being a third type of trophoblast: the “inner cells” (revised in Wislocki and Dempsey, 1946). They also stated that the ECM that set those cells off from the trophoblast was continuous with the collagenous matrix of the subjacent mucosa (Wislocki and Dempsey, 1946). The stromal origin of those cells was later confirmed by immunohistochemical analysis of the intermediate filaments’ proteins (section 2.1). Subsequent research allowed gaining knowledge into morphological and functional characteristics of feline decidual cells. Besides the domestic cat, these cells have been also found in Iberian lynxes (*Lynx pardinus*) (Braun et al., 2012a). According to Srivastava (1952), DSCs are not found in the placenta of the tiger (*Panthera tigris tigris*).

**FIGURE 2:**
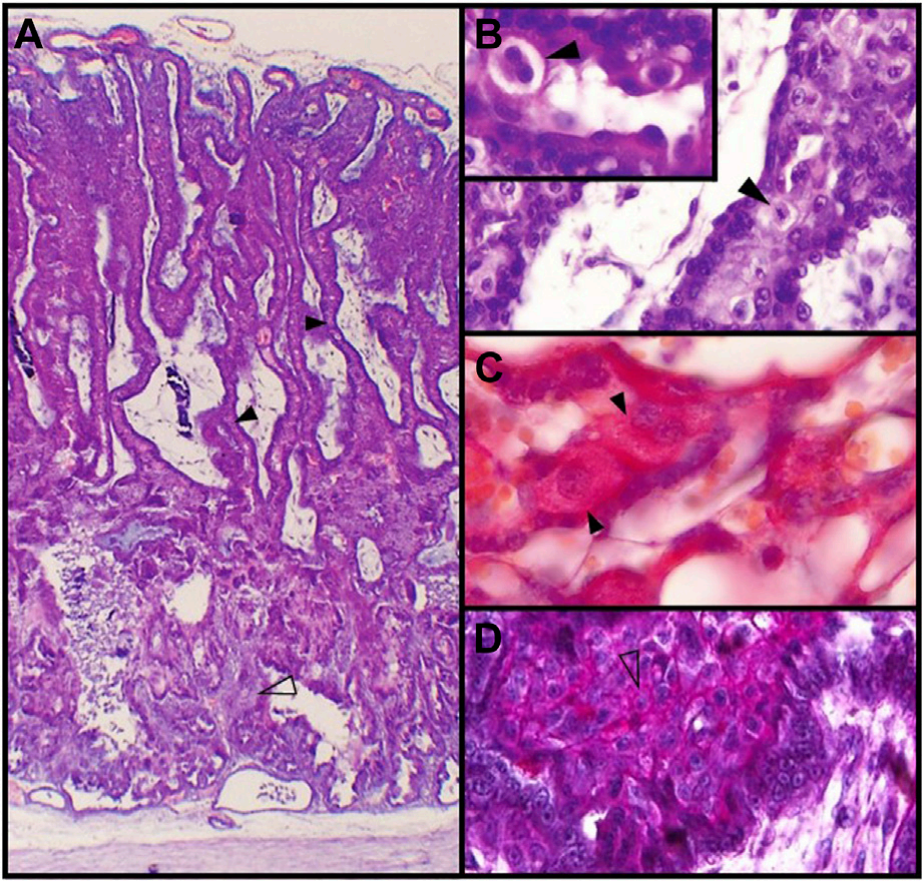
Feline placenta. **(A)**. Structure of the feline placenta. HE. Laminar arrangement of maternal and fetal structures (4X). **(B)**. Labyrinth. The black arrowhead points to a mitotic figure in a DSC. HE. (40X). Inset: prominent ECM surrounding a binucleated DSC (100X). **(C)**. Labyrinth. Mono- and binucleated labyrinthine DSCs. Periodic acid-Schiff (PAS) method (100X). **(D)**. Plaque of DSCs at the junctional zone. Alcian blue-PAS technique (40X) (Diessler et al., 2014). Black arrowheads: DSCs; empty arrowheads: small DSCs.

**FIGURE 3:**
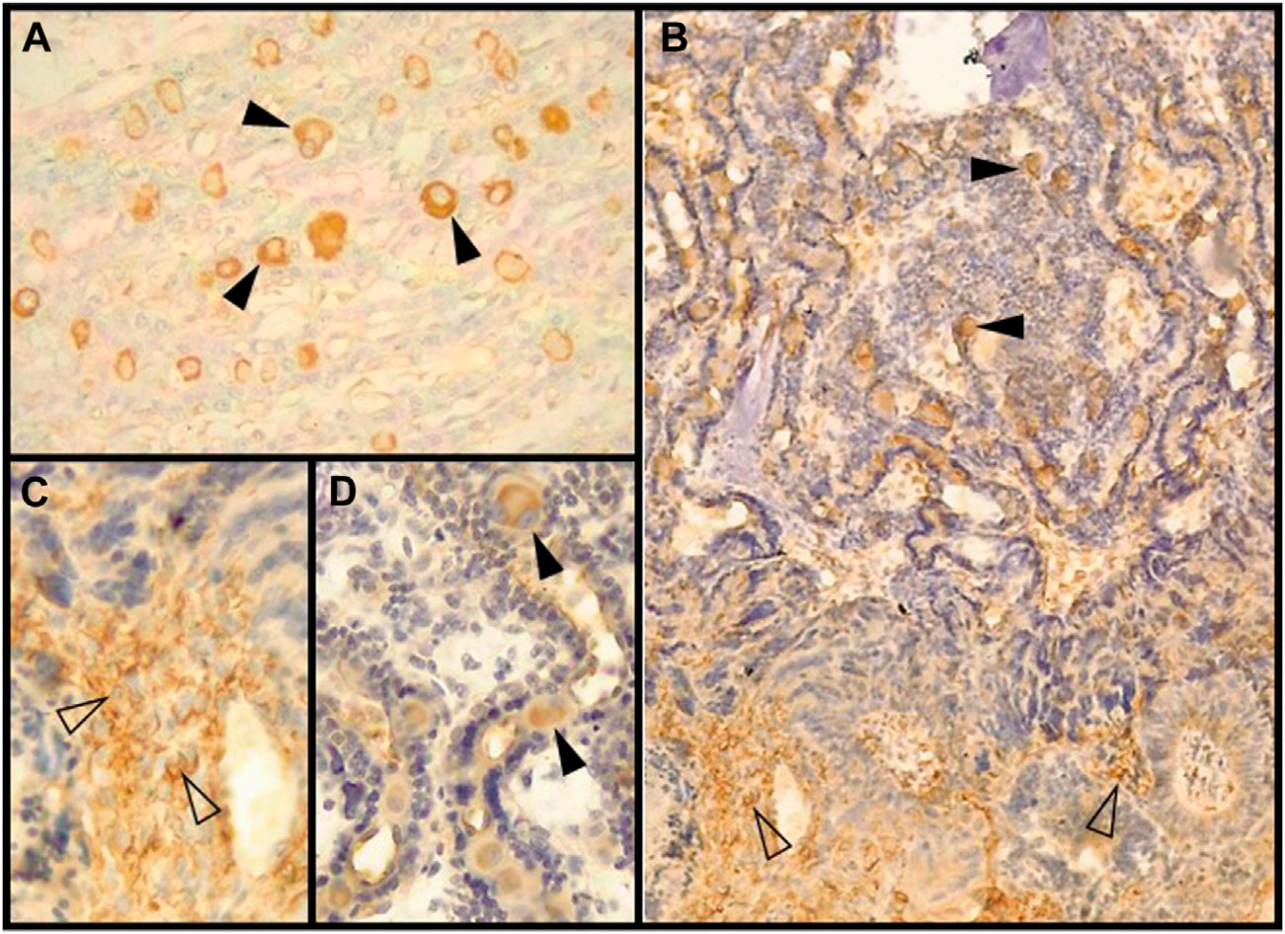
Decidual stromal cells (DSCs) in the feline placenta. Immunohistochemical staining of cytoskeletal proteins. **(A)**. Vimentin-positive DSCs in the labyrinth (40X) (Clone V9, RTU, Code: IS630. Dako). **(B)**. α-SMA (Code: NCL-L-SMA. Novocastra) (4X). Black arrowheads: DSCs in the lamellae; empty arrowheads: small DSCs at the junctional zone (JZ). **(C, D)** magnification of **(B)**. **(C)**. Plaque of DSCs at the JZ (40X). **(D)**. Labyrinth (40X). IHC. DAB. Hernández et al., 2017.

The appearance of recognizable decidual cells in the queen´s placental junctional zone (JZ) is not described until post implantation stages, although changes in the ECM quality are obvious before morphological evidence of cell differentiation (Boomsma et al., 1991; Walter and Schönkypl, 2006; Diessler et al., 2014). Stromal cells in the JZ, beginning their morphological differentiation, have been mentioned as “predecidual” (Amoroso, 1959; Boomsma et al., 1991; Leiser and Koob, 1993). Their cytoplasm is pale and more or less abundant and they are arranged in groups, forming a compact area or *plaque*, which is set out as a palisade between the labyrinth and the glandular “spongy” zone (Amoroso, 1959; Leiser and Koob, 1993; Diessler et al., 2014) (Figures 2A, D; Figures 3B,C). This area shrinks as gestation advances and DSCc are gradually incorporated into the labyrinth, where they are lodged between maternal capillaries within the lamellae (Wislocki and Dempsey, 1946; Amoroso, 1959; Leiser and Koob, 1993; Wooding and Burton, 2008). Once in the lamellae, DSCs are displayed first in rows and then become conspicuously larger and solitary, constituting the typical giant cells; their number decreases with time (Amoroso, 1959; Malassiné A, 1974; Walter and Schönkypl, 2006). In the last years, while some authors name as decidual the entire cell population (the more or less differentiated cells), others refer to larger cells within the lamellae as “giant cells” and reserve terms as “decidual” or “decidualized” for those in the JZ plaque. In this review, unless otherwise stated, the former option is adopted; therefore, cells in the JZ plaque and in the lamellae will be referred to as DSCs.

During their development, DSCs are oval to round, vary from 15 to 50 µm, and have one to three spherical and increasingly euchromatic nuclei, with well-defined nucleoli (Wislocki and Dempsey, 1946; Dempsey and Wislocki, 1956; Malassiné, 1974; Wooding and Burton, 2008). Nearly 20% of the DSCs are binucleated (Fernández et al., 2014), and mitotic figures are often seen, although there are not available records of telophase figures or cleavage furrows (Wislocki and Dempsey, 1946; Walter and Schönkypl, 2006) (Figures 2B, C). Therefore, binucleation is likely to occur as a consequence of truncated cytokinesis rather than of cell fusion. Varying degrees of polyploidy, with or without binucleation, has been reported in decidual cells in mice and human, in physiologic conditions (Sroga et al., 2012; Peterson and Fox, 2021). Those cells become polyploid as a result of either a switch from a mitotic cycle to an endoreduplication cycle, or, being endomitotic, the progression to anaphase and then an incomplete cytokinesis. Decidual polyploid cells are considered to be terminally differentiated (revised in Sroga et al., 2012). Multinucleate polyploid cells may allow slightly larger cell size than equivalently polyploid mononucleate cells, and maintain the nuclear-cytoplasmic ratio in spite of their hypertrophy conditions (Sroga et al., 2012; Peterson and Fox, 2021). As regards the carnivore placenta, polyploidy has been described only in trophoblast cells in the silver fox (Zybina et al., 2001).

Ultrastructurally, two zones can be recognized in the cytoplasm: a dense, eccentric zone around the nucleus where membranous organelles are abundant, being the endoplasmic reticulum particularly well-developed, and a peripheric one almost entirely free of organelles (Wislocki and Dempsey, 1946; Wynn and Björkman, 1968; Malassiné A, 1974). Some dense bodies described as “continuous with the extracellular matrix” were described (Malassiné A, 1974). Cytosolic inclusions as lipidic droplets and the so called “glycogen bodies” are present in DSCs and increase with time; microvilli extend towards the endothelial cells, and through the ECM towards the STB (Malassiné A, 1974).

Several molecules were targeted to feline DSCs; some of those findings are mentioned or discussed below, and summarized in Table1.

##### 2.3.1.2 Extracellular matrix in the labyrinth

ECM surrounds DSCs as a capsule and shows positivity for Periodic acid-Schiff (PAS) method and type IV collagen (Figures 2B, C); this matrix is partially continuous, though thinner, with the basal lamina (or “interstitial membrane”) of maternal vessels (Dempsey and Wislocki, 1956; Wynn and Björkman, 1968; Malassiné A, 1974; Leiser and Koob, 1993; Walter and Schönkypl, 2006; Diessler et al., 2014). The presence of reticular fibers might be inferred from the silver deposits around DSCs (Wislocki and Dempsey, 1946); however, by fluorescent immunohistochemistry, Walter and Schönkypl (2006) could not detect type III collagen within the maternal lamellae. On the other hand, laminin, which is a decidualization marker, was targeted to decidual cells, both within the labyrinth and in the junctional zone. In placentas from early gestation, MMP-2 was identified in DSCs, while MMP-1, -9 and -13 were not (Walter and Schönkypl, 2006).

##### 2.3.1.3 Glycosylation patterns and galectins

The glycocode of a cell constitutes a kind of biological information, spatiotemporally regulated. It has been demonstrated that glycosylation pattern of decidual cells differ among species; for instance, sialic acids and highly branched N-linked oligosaccharides, which characterize mice and rats DSCs, were not demonstrated in cats (Bulmer and Peel, 1996; Jones et al., 1996; Fernández et al., 2014). This differential pattern might obey to both a species-specific glycan expression and distinct maternal-fetal interfaces (placental barrier types). In addition, residues forming the oligosaccharides also change during DSCs feline differentiation: while mature cells already express α-GlcNac as well as mannose and fucose in their oligosaccharides, still undifferentiated stromal cells do not (Fernández et al., 2014). Besides through growth and differentiation, in which glycans have crucial roles, changes in glycosylation pattern were reported likewise as a feature of the cellular response to microenvironmental challenges, e.g., changes in redox imbalance (Blois et al., 2021; Menkhorst et al., 2021).

**FIGURE 4:**
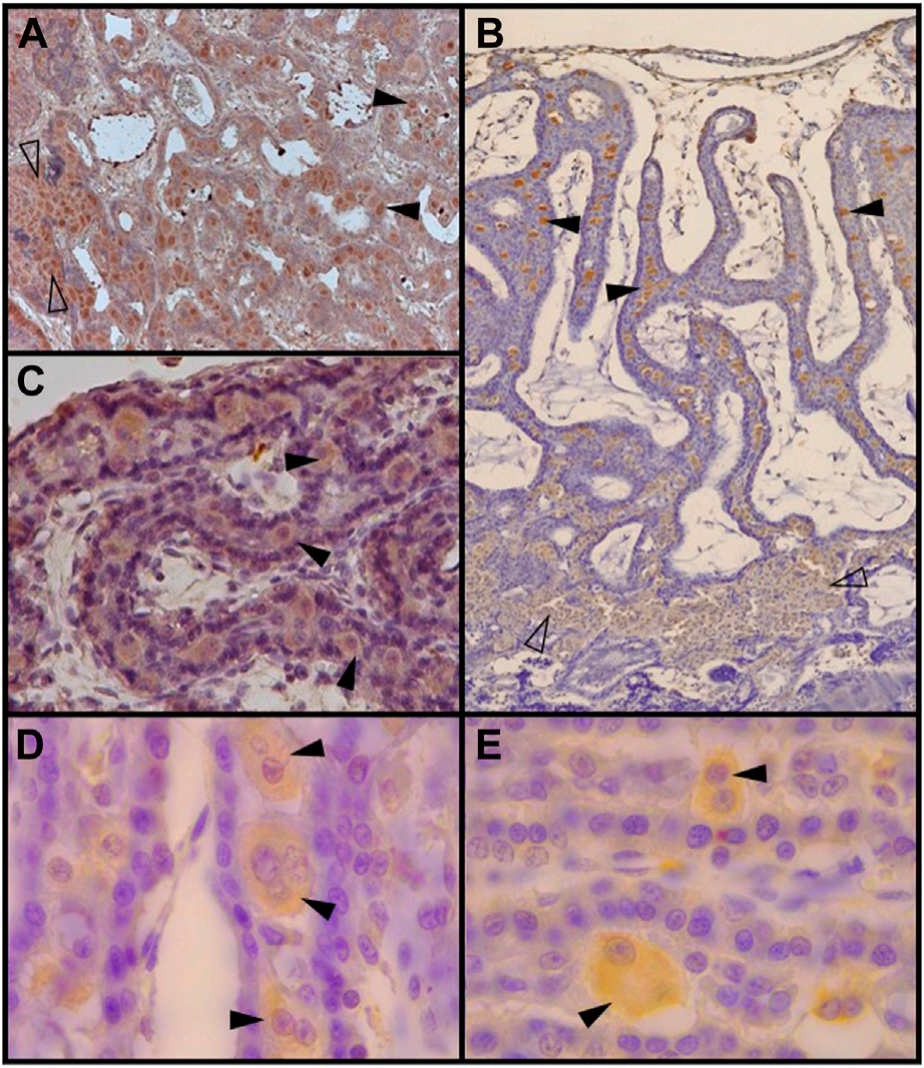
Decidual stromal cells (DSCs) in the feline placenta. Immunohistochemical staining of: **(A)**. Galectin-9 (Code: sc-19292. Santa Cruz Biotechnology, Diessler et al., 2014) (10X), **(B)**. VEGFR-2 (Clone 55B11, Code: 2479S. Cell Signaling Technology, Gomez Castro et al., 2022a) (4X), **(C)**. IGFBP-1 (40X) (Code: PAAH1. Novozimes GroPep Ltd., Hernández et al., 2017), **(D)**. Prolactin (100X), and **(E)**. Prolactin receptor (100X). Black arrowheads: DSCs in the labyrinth; empty arrowheads: small DSCs at the junctional zone (Codes: AB186522 and AB2772. Abcam, Hernández et al., 2019). IHC. DAB.

Members of the galectin family are glycan binding proteins, regarded as important dynamic translators of the glycocode. They influence signaling processes cross-linking glycans, mainly membrane bound (Wisnovsky and Bertozzi, 2022). Alternatively, they may be translocated to the nucleus and participate in mRNA splicing (Than et al., 2012). Galectin research become relevant to reproductive sciences as these proteins are highly expressed at the maternal–fetal interface, and their dysregulated expression is observed in the ‘great obstetrical syndromes’ in human beings (Than et al., 2012; Menkhorst et al., 2021). During pregnancy, galectins are involved mainly in the regulation of angiogenesis and contribute to the development of an immune-privileged environment at the maternal-fetal interface; their expression in the endometrium and decidua is strictly regulated by sex steroids. Galectins 1, 3 and 9 are the main galectins expressed in endometrial and decidual cells (reviewed in Than et al., 2012; Menkhorst et al., 2021). Gal 1and gal 3 are involved in the downregulation of local inflammatory pathways; in the maternal-fetal human interface, their expression decreases during labor (El-Azzamy et al., 2017). Gal-1, -3 and -9 were immunolabeled in the feline placenta; they were targeted to DSCs, besides being also strongly positive in trophoblast cells (Conrad et al., 2016). The stronger labeling of galectins in DSCs corresponded to that of Gal-9 (Figure 4A). Regarding temporal modifications in galectin detection, Gal-1staining was markedly increased from earlier to later placentas. An according significant change in *LGALS1* (gene for Gal-1) was reported in humans, being *LGALS1* and *IGFBP1* the two highest expressed genes in the decidua at term (El-Azzamy et al., 2017). In addition to changes regarding staining intensity, another remarkable finding was that Gal-1 and Gal-9 were detected not only in the cytoplasm but also in the nuclei in late placentas. This finding suggests that they might exert a role in mRNA processing; this shift could be related to changes in hormone profiles at the time. All in all, decidual galectin expression appears to be lower in endothelial placentas than in more invasive hemochorial placentas (of mice and humans, e.g.,) where immunotolerance is, to some extent, more required for a successful pregnancy (Conrad et al., 2016).

##### 2.3.1.4 Progesterone synthesis

Several questions have arisen for decades regarding steroidogenesis in the feline placenta: its capacity to produce steroids as progesterone (*de novo* or from pregnenolone), the source of intermediate molecules, the contribution of placental P4 to progesteronemia, the cell source of the placental progesterone and, finally, the sufficiency of such progesterone to support pregnancy in the event of ovariectomy. Although some data was gathered to enlighten such issues (including reports of variable proportions of successful pregnancies after ovariectomy) a number of those controversies remains unsolved.

It was stated that the feline placenta meets the basic requirement for the last step in steroid biogenesis, as 3β-HSD activity (leading to conversion of pregnenolone to progesterone) was detected (Malassiné and Ferré, 1979). Later on, the histochemical distribution of 3β-HSD was described (Ito et al., 1986), and mRNA expression of several steroidogenic enzymes, including 3β-HSD, and detectable levels of P4 and E2 in placental tissues were reported (Braun et al., 2012b). Total enzymatic activity was shown to be clearly increased during the second half of pregnancy, specially from 40 to 45 dpc (Malassiné and Ferré, 1979; Braun et al., 2012b). Nevertheless, at that stage, peripheral concentration of placental P4 is extremely low or even lies below the detection threshold, therefore it is assumed that placental P4 does not influence serum P4 levels (Braun et al., 2012b). Taking into account that notion, a paracrine role in supporting placental function, if such, may well be assumed; this influence in pregnancy success might be only significant after 40 dpc (Tsutsui et al., 2009). However, neither ER nor PGR were detected in the placenta by Li et al. who studied placentas up to 28 dpc (Li et al., 1992).

The finding of STAR mRNA and its protein in feline placentas allowed inferring that P4 may potentially be synthesized *de novo* from cholesterol in this species, as it regulates cholesterol transfer within the mitochondria, and this is the rate-limiting step in steroidogenesis (Siemieniuch et al., 2012). As far as the role of feline decidual cells in steroidogenesis or steroid signaling is concerned, while Ito et al. concluded, based on a histochemical study, that enzymatic activity of 3β-HSD was restricted to trophoblast cells, Siemieniuch et al. (2012) reported that immunolabeling was localized to decidual cells.

##### 2.3.1.5 Growth factors (GFs)

Besides steroid hormones, and among other substances, growth factors and their receptors are required for the establishment and progression of pregnancy (Guzeloglu-Kayisli et al., 2009). It is considered that EGF/TGFα/EGFR system is upregulated during decidualization and are involved in the regulation of proliferation and differentiation in diverse placental cell populations in baboons (Fazleabas et al., 1994). TGF is involved in trophoblast differentiation and might also, through its downstream target Kisspeptin, have a role in decidualization and in restraining trophoblast invasion (Cao et al., 2019; Fang et al., 2022). EGF, TGFα and EGFR have been immunohistochemically studied in the feline placenta. While EGF and TGFα were demonstrated in the syncytiotrophoblast until the 6^th^ week pc, from 24 dpc onwards they were targeted to decidual cells. Interestingly, the receptor (which binds both ligands) was detected in the trophoblast cells for a short period, then it was stained both in cytotrophoblast and decidual cells, and finally it was restricted to decidual cells (from the 7^th^ week onwards) (Fazleabas et al., 1994; Boomsma et al., 1997). From these results, it could be inferred that until the 6^th^ week of gestation, autocrine and paracrine signaling take place; afterward, signaling goes on, by an autocrine mechanism, only in decidual cells.

Taken together with the lack of steroid receptors during almost the first half of gestation, the finding of reactivity to TGF and EGF led to the speculation that, at that stage, development and maintenance of the placenta might depend more on GFs than on sex steroids (Fazleabas et al., 1994; Boomsma et al., 1997). Almost a decade after, several studies about pregnancy termination using PGR inhibitors began to be conducted by different research groups. As a result, induced abortion was observed after treatment with aglepristone in early and mid-pregnancy, showing the crucial role of progesterone in gestation maintenance (Fieni et al., 2006; Georgiev and Wehrend, 2006; Georgiev et al., 2010; García Mitacek et al., 2012). Therefore, further studies on placental PGR detection and localization become necessary.

IGF/IGFBP-1/IGF1R is another system essential for placentation, and particularly, for decidualization of the uterine stroma. IGFBP-1 is considered, together with prolactin, a major decidual marker in humans and baboons (Fazleabas et al., 1989; Dunn et al., 2003; Tang et al., 2005). IGFBP-1 was evidenced by culture medium immunoblotting from feline placental explants; additionally, it was localized to DSCs (Boomsma et al., 1994; Hernández et al., 2017) (Figure 4C). To a lesser extent, in a preliminary study, it was also detected in the endometrial fibroblasts in close proximity to small decidual cells in the JZ plaque (Hernández et al., 2017). In another initial analysis, Hernández et al. reported the detection in DSCs of the other major decidualization marker, prolactin, as well as its receptor (Hernández et al., 2019) (Figures 4D, E).

VEGF system plays a crucial role during decidual angiogenesis through the action of VEGFR-2 (Douglas et al., 2009). Feline DSCs, together with the syncytiotrophoblast, were strongly labeled for VEGF-A in placentas at different stages of pregnancy. Its main receptor, VEGFR-2, showed an irregular staining in DSCs of early placentas (from negative to strongly positive), becoming consistently positive in the later ones in those cells (Figure 4B); conversely, it was regularly detected throughout pregnancy in the syncytiotrophoblast (Gomez Castro et al., 2022a). This spatial-temporal pattern in a way resembles that of EGF, TGFα and EGFR, which reflects an increasing involvement of decidual cells in these pathways.

##### 2.3.1.6 Relaxin

The first studies about relaxin (RLN) in the feline placenta were conducted by Dr. Stabenfelt´s research group in the ´80s. Based on their results, it was established that relaxin is a pregnancy-specific signal in feline species (Stewart and Stabenfeldt, 1985; Addiego et al., 1987). Lacking a species-specific antibody, those studies were performed using antisera developed against porcine relaxin which, at that time, allowed determining the source of the hormone and providing a profile of its secretion along pregnancy. RLN immunoactivity greatly increased by day 28 pc, and the higher amount was detected on day 35 pc; in later stages, although still high, immunoreactivity was lower than on previous days (Stewart and Stabenfeldt, 1985; Addiego et al., 1987).

Klonish et al., in 1999, after performing ISH and IHQ on cryocut sections of a 35 dpc placenta, reported that the sole source of RLN and relaxin mRNA transcripts were the trophoblast cells in the labyrinth (Klonisch et al., 1999). Later on, Braun et al. reported positivity for RLN not only in the trophoblast cells but also in fetal vessels and in DSCs, both in cats and in placentas from two Iberian lynxes (*Lynx pardinus*) (Braun et al., 2012a). It has been reported that this apparently decidual product is useful as a pregnancy marker in cats, in the leopard (*Panthera pardus*) (de Haas Van Dorsser et al., 2007) and in the Iberian lynx (Braun et al., 2012a).

##### 2.3.1.7 Orexin and leptin

According to immunohistochemical studies performed in feline placentas from 55 to 60 days, DSCs were strongly stained for orexin-A (OXA), leptin (LEP, also Ob), and their receptors (OX1R and LEPR, respectively); OX2R, on the other hand, was not detected (Dall’Aglio et al., 2012b; Dall’Aglio et al., 2012a). STB was also positive, though inconsistently, to the same substances; this finding supports that the neuropeptide orexin and leptin protein participate in the paracrine dialogue between neighboring cells in the lamellae. The detection of OXA, OX1R, LEP, LEPR in the feline placenta might imply that this organ is a peripheric source and target in both systems. OXA and LEP have been also detected in human placental cells (Masuzaki et al., 1997; Nakabayashi et al., 2003). It was reported that orexin inhibits *LEP* expression in mice *in vivo* and *in vitro* (Shin et al., 2019). Their physiological functions outside the brain and the adipocytes, and so their role in pregnancy, remain poorly defined.

##### 2.3.1.8 Prostaglandin synthesis

In early feline placentas, PGFS protein and transcripts were elevated, being localized mainly in trophoblast cells; later on, it was also detected in decidual cells. Conversely, *PTGS2* was only upregulated in placentas from queens close to term. PTGS, the enzyme catalyzing PGH2 synthesis, was located in trophoblast as well as in decidual cells during the last week before labor. PGH2 can be regarded as the precursor of all other prostaglandins, beside other molecules. PGF2α (and PGFM, its stable metabolite) was increased in late placentas, despite the decrease in PGFS, the enzyme that catalyzes its production. This discrepancy might obey to the eventual production of PGF2α through other pathways (Siemieniuch et al., 2014). PGFM was studied in fecal samples of eighteen felids using enzyme immunoassay to establish its usefulness for the differentiation between pregnancy and pseudopregnancy in captive and free-ranging felids, that is, as a non-invasive pregnancy marker. Animals from the lineages of domestic cat, leopard cat, puma, lynx, ocelot, caracal, and panthera (based on phylogenetic analyses in Johnson et al., 2006) were sampled. Although there were some discrepancies among the species of the lineage Panthera, all in all fecal detection of PGFM proved to be a useful tool (Dehnhard et al., 2012). It is not known to what extent the decidual metabolite could take part in the amount of PGFM detected by the test.

##### 2.3.1.9 Transporters

Glucose transporters (GLUTs) and aquaporins (AQPs) were studied in the cat placenta. As far as GTs are concerned, Ferré-Dolcet et al. reported that not only GLUT1 but also GLUT3 is found in the feline labyrinth, unlike what was stated by Wooding et al. some years before. Besides, it was demonstrated a negative correlation between GLUT3 and P4 levels in the queen. Although DSCs storage of glycogen is thought to play a role in carbohydrate metabolism in the placenta, neither of GTs was located in those cells (Wooding et al., 2007; Ferré-Dolcet et al., 2018). Neither AQP2 nor 8 was detected in DSCs, although they did were demonstrated in trophoblast cells (Ferré-Dolcet et al., 2020).

## 3 Discussion

Development of decidual cells is a distinct feature of eutherian pregnancy; however, the occurrence of decidualization itself, the dynamics of its development, as well as morphological and functional features of DSCs vary widely among eutherians. Within the carnivores known to develop endotheliochorial placentas, decidual cells could be evidenced only in a few species.

Placentas of dogs and cats have been more thoroughly studied than those of any other carnivore (Mossman, 1987; Wooding and Burton, 2008); from the majority of the remaining species of the order, data in the bibliography are fragmentary. This may be due to lesser availability of individuals, sampling difficulties -sometimes in challenging circumstances-, and the eventual inadequate preservation of tissues. In addition, placentophagy, the “consumption” of the placenta after birth in different animals, is especially widespread in carnivores (Benirschke, 2011). Moreover, most of the reports in wild carnivores precede the advent of immunohistochemical techniques. The *in vitro* decidualization of canine uterine stromal cells attained by Kautz et al. (2015), and the establishment of an immortalized cell line achieved by Graubner et al. (2017) have boosted research on canine DSCs. More recently, the promising development of a 3D culture of feline endometrial cells was reported (Dundon et al., 2019). In this *in vitro* platform, the effects of E2 and P4 at physiologically relevant concentrations on endometrial cells began to be tested, and results regarding epithelial cells were documented (Wilsterman et al., 2019).

Up to now, fairly abundant data has been collected regarding the bitch and the queen DSCs. Only IGFBP-1, among the main decidual markers, has been undoubtedly demonstrated in both species. Laminin, on the contrary, was found only in feline DSCs, and prolactin was evidenced in a preliminary report in dogs and cats, requiring further studies. Prolactin receptor, on the other hand, was found in both species.

It is currently accepted that P4 synthesis does not occur in the bitch placenta; conversely, P4 might be produced in the feline placenta. However, while canine DSCs are the only placental cell type expressing PGR, these receptors have not been demonstrated neither feline DSCs, nor in any other cell in the queen placenta. Striking question arise from the apparently proven facts that placental P4 does not contribute to serum P4 (Braun et al., 2012b), and that PGRs have not been found up to now in the feline placenta (Li et al., 1992). From this view, receptors required for a local communication would be lacking. As Li et al. studied placentas up to 28 dpc, the analysis of samples from older pregnancies could shed light on the matter. Furthermore, as the use of PGR inhibitors leads to abortion both in early and in mid-gestation (Fieni et al., 2006; Georgiev and Wehrend, 2006; Georgiev et al., 2010; García Mitacek et al., 2012), more exhaustive studies in this regard become essential. In addition, progesterone might act by receptor-independent pathways and, as it is mentioned above (section 2.3.1.5), during the early pregnancy, the development and maintenance of the placenta might be at least in part also supported by GFs.

Growing knowledge about, for instance, specific angiogenic mediators and other growth factors, metalloproteases, galectins, glycans, and so forth, will allow to achieve a deeper and substantial understanding of vascularization and remodeling of the endotheliochorial interface during placental organogenesis. Most of the analyses reviewed in this article are novel for domestic animals, and comprehensive studies of a higher number of placentas are needed to establish the consistency of the results, particularly to progress on the characterization regarding some aspects of the DSCs their selves, and also of decidualization as a process. Besides, placentas from different stages of pregnancy will enable researchers to establish how molecules change with time and to robustly speculate about the physiologic significance of the findings.

From the data reviewed, it follows that carnivoran DSCs take part either in the secretion of progesterone, prostaglandins, relaxin, among other substances, or at least in the signaling pathways triggered by them. Beyond their physiological roles, some of those molecules are already being used, or are yet under study, for the non-invasive endocrine monitoring and reproductive control of domestic and wild carnivores. For instance, relaxin is used as the active principle of canine and feline ELISA pregnancy tests. Besides its use for companion carnivores, it is being tested as a managing tool in conservation breeding programs for endangered wild felids, as the Iberian lynx (*Lynx pardinus*) and the Arabian leopard (*Panthera pardus nimr*) (de Haas Van Dorsser et al., 2006; de Haas Van Dorsser et al., 2007; Harris et al., 2008; Vargas et al., 2008; Jewgenow and Songsasen, 2014). It has also been successfully tested in the following wild canids: gray wolves (*C.lupus*), Mexican gray wolves (*C. l. baileyi*), red wolves (*C. rufus*), fennec foxes (*Vulpes zerda*), African wild dogs (*Lycaon pictus*), island foxes (*Urocyon littoralis*) (Bauman et al., 2008), and coyotes (*C. latrans*) (Carlson and Gese, 2007). Measuring of progestogen metabolites has been studied for monitoring the reproductive status in the cheetah (*Acinonyx jubatus*) (Koester et al., 2017). In addition, the usefulness of PGF2α metabolite PGFM for testing pregnancy has been studied in felid species from the lineages of domestic cat, leopard cat, puma, lynx, ocelot, caracal, and panthera (Dehnhard et al., 2012).

Pharmacological termination of pregnancy for the control of reproduction has been a growing field of research in veterinary medicine. Drugs intending to block different molecular targets that support pregnancy are used in bitches and queens, and the success of the clinical procedure relies on several factors (Fieni et al., 2006; Georgiev and Wehrend, 2006; Georgiev et al., 2010; García Mitacek et al., 2012; Karakas Alkan et al., 2020; Kowalewski et al., 2020; Binli et al., 2022). Data regarding the sources (including decidual contribution) of relevant substances, namely, progesterone, prolactin, and prostaglandins, and the understanding of their functions and dynamics of secretion along pregnancy, are required for the improvement of those methods.

## Footnotes

1 Given the wide differences regarding relaxin aminoacidic sequence among species, it is worth mentioning that in the referred work immunohistochemistry was conducted using canine-specific, custom-made antibodies.
