# Supplementary material for: Decidual cells and decidualization in the carnivoran endotheliochorial placenta
Source: Front Cell Dev Biol. 2023 Mar 16;11:1134874. doi: 10.3389/fcell.2023.1134874 (PMC10060884; doi:10.3389/fcell.2023.1134874)
Supplement: Supplementary file 1 [file Table1.DOCX]

**Supplementary material**

Table. Reagents used for the indirect IHC analyses mentioned in the figures' captions. DAB was used as chromogen in each case

α-SMA: smooth muscle actin; DAB: 3,3-diaminobenzidine tetrahydrochloride; IGFBP-1: insulin-like growth factor binding protein 1; PRL: prolactin; PRLR: prolactin receptor; RTU: ready to use; VEGF‑R2: vascular endothelial growth factor receptor 2.

| **Antibody against** | **Type of antibody** | **Clone** | **dilution/**  **RTU** | **Cat. N°** | **Company** | **Detection system/company** |
| --- | --- | --- | --- | --- | --- | --- |
| **α-SMA** | monoclonal mouse | αsm-1 | 1/50 | NCL-L-SMA | Novocastra | EnVision™ System and DAB (Invitrogen™) |
| **desmin** | monoclonal mouse | D33 | RTU | IS606 | Dako | EnVision™ System and DAB (Invitrogen™) |
| **Galectin‑9** | polyclonal goat |  | 1/100 | sc-19292 | Santa Cruz Biotechnology | Donkey anti-goat PO-conjugated secondary Ab (1:200, cat. #705-035-147 Jackson ImmunoResearch) |
| **IGFBP-1** | polyclonal rabbit |  | 1/500 | PAAH1 | Novozymes GroPep Ltd. | CytoScan™ Cell Marque HRP, cat. #  [CMC951080030](https://www.medical-supply.ie/product/cytoscan-hrp-detection-system/) |
| **PRL** | polyclonal rabbit |  | 1/100 | AB186522 | Abcam | Dako EnVision+ System- HRP Labelled Polymer  Anti-Rabbit™ |
| **PRLR** | monoclonal mouse | U5 | 1/100 | AB2772 | Abcam | Dako EnVision+ System- HRP  Labelled Polymer  Anti-mouse™ |
| **VEGF-R2** | monoclonal rabbit | 55B11 | 1/200 | 2479S | Cell Signaling Technology | CytoScan™ Cell Marque HRP, cat. #  [CMC951080030](https://www.medical-supply.ie/product/cytoscan-hrp-detection-system/) |
| **vimentin** | monoclonal mouse | V9 | RTU | IS630 | Dako | EnVision™ System and DAB (Invitrogen™) |
